# Global and regional trends of people living with HIV aged 50 and over: Estimates and projections for 2000–2020

**DOI:** 10.1371/journal.pone.0207005

**Published:** 2018-11-29

**Authors:** Christine S. Autenrieth, Eduard J. Beck, Dominik Stelzle, Christoforos Mallouris, Mary Mahy, Peter Ghys

**Affiliations:** 1 UNAIDS, Programme Branch, Geneva, Switzerland; 2 UNAIDS, Latin American and Caribbean Regional Support Team, Georgetown, Guyana; British Columbia Centre for Excellence in HIV/AIDS, CANADA

## Abstract

**Background:**

The increasing numbers of people living with HIV (PLHIV) who are receiving antiretroviral therapy (ART) have near normal life-expectancy, resulting in more people living with HIV over the age of 50 years (PLHIV50+). Estimates of the number of PLHIV50+ are needed for the development of tailored therapeutic and prevention interventions at country, regional and global level.

**Methods:**

The AIDS Impact Module of the Spectrum software was used to compute the numbers of PLHIV, new infections, and AIDS-related deaths for PLHIV50+ for the years 2000–2016. Projections until 2020 were calculated based on an assumed ART scale-up to 81% coverage by 2020, consistent with the UNAIDS 90–90–90 treatment targets.

**Results:**

Globally, there were 5.7 million [4.7 million– 6.6 million] PLHIV50+ in 2016. The proportion of PLHIV50+ increased substantially from 8% in 2000 to 16% in 2016 and is expected to increase to 21% by 2020. In 2016, 80% of PLHIV50+ lived in low- and middle-income countries (LMICs), with Eastern and Southern Africa containing the largest number of PLHIV50+. While the proportion of PLHIV50+ was greater in high income countries, LMICs have higher numbers of PLHIV50+ that are expected to continue to increase by 2020.

**Conclusions:**

The number of PLHIV50+ has increased dramatically since 2000 and this is expected to continue by 2020, especially in LMICs. HIV prevention campaigns, testing and treatment programs should also focus on the specific needs of PLHIV50+. Integrated health and social services should be developed to cater for the changing physical, psychological and social needs of PLHIV50+, many of whom will need to use HIV and non-HIV services.

## Introduction

Populations are ageing across the world due to rising life expectancy and falling fertility rates, which has led to the adoption of the first Global Strategy and Plan of Action on Ageing and Health by the World Health Assembly in 2016 [1]. Similarly, the number of people living with HIV aged 50 years and over (PLHIV50+) is increasing [2–5]. Some of these acquired HIV when they were aged less than 50 years and were treated with antiretroviral therapy (ART), while others acquired HIV when 50 years or older. For a successful HIV response, the number of people acquiring HIV and the number of people dying of AIDS should both decline.

Evidence suggests that PLHIV develop non-HIV health conditions at an earlier age than people not living with HIV, [6–10], and that these non-HIV health conditions are severe [11–16]. Apart from HIV infection and long-term ART, this accelerated and accentuated ageing can be due to other factors, including socio-economic and lifestyle factors such as tobacco use, physical inactivity, excessive alcohol intake and poor nutrition. For women living with HIV, this includes health conditions related to menopause and reduced bone density [17–19]. Ageing with HIV can also have mental health impacts, due in part to stigma and discrimination [20] while depression can hamper the adherence to ART [21]. ART is currently therapy for life, and the long-term adherence to and side-effects of ART will need to be better explored [22–24].

Data on PLHIV50+ are essential to better understand the health and social needs of this age group, and to develop and implement appropriate health and social services [25]. However, such data are still scarce in countries with high HIV prevalence; data collection in population-based household surveys have mostly focused on 15–49 year old persons and on pregnant women attending antenatal clinic facilities. It is also important for people aged over 50 to understand the risks of becoming infected with HIV and to improve prevention activities in this population.

The aim of this paper is to present the current global and regional HIV burden among PLHIV50+ based on the 2017 UNAIDS estimates that cover the period 2000–2016, to project estimates up to 2020 and to review the treatment and care services, prevention and social services that countries need to develop and implement to cater for their increasing number of PLHIV50+ and to reduce incidence among people aged older than 50 years.

## Methods

### Historical and current HIV estimates

UNAIDS supports countries to produce models on an annual basis of the impact of HIV on their population [26,27]. These models allow country teams to estimate the impact of HIV and the underlying demographic changes in their population. The Spectrum model [28], which is used to develop these estimates, is based on the demographic structure of a country and progresses that population through time considering fertility, mortality and migration data from the UN Population Division’s World Population Prospects (WPP) [29]. In the 2017 model, the WPP 2015 estimates were used to inform the demographic variables. The Spectrum model Spectrum version 5.57 was used to calculate HIV estimates of PLHIV50+ for the present analysis.

Using the basic demographic input in the model, the AIDS Impact Module within Spectrum is further informed by HIV prevalence data, obtained from sentinel surveillance surveys among women attending antenatal clinics, surveys among key populations, household surveys that include HIV serostatus, case reporting data, as well as program data on the number of PLHIV reached with ART. The best available surveillance data, such as case surveillance where those data are of high quality, are entered into the model to ensure the modelled results reflect the available data.

Most of these surveillance activities are focused on women and men aged 15–49 years. Using the historical prevalence data, the model then estimates incidence taking into consideration ART coverage over time. The AIDS Impact Module is not a deterministic model, so information on interventions do not have an important impact on the incidence estimates as those are derived from the prevalence from surveillance and the ART coverage. Incidence is distributed by age and sex for the entire population based on assumptions on incidence rate ratios of the incidence among people 25–29 to all age groups from 15–19 through 80+. These ratios are primarily based on data available from longitudinal cohort studies in different epidemic settings. For high prevalence epidemic countries, the data are derived from demographic and surveillance sites in Eastern and Southern Africa. For low level epidemics with few people who inject drugs the patterns of incidence by age and sex are derived from population-based household surveys in countries with HIV prevalence less than four percent. Finally, in the low level epidemics with more than fifty percent of new infections through people who inject drugs, the incidence distribution comes from studies in the former Soviet Union [30,31].

AIDS-related mortality among people not receiving ART comes from studies prior to the expansion of ART. AIDS-related mortality among people receiving ART is derived from the International Epidemiology Databases to Evaluate AIDS (IeDEA) [32]. Regional averages for these parameters are derived from the IeDEA sites in each region, including a set of assumptions for developed countries. The IeDEA consortium does not include European sites thus the parameters for developed countries are based on IeDEA sites in the United States, Canada and other high-income countries. Among people who inject drugs, an additional mortality multiplier is added to reflect the additional non-AIDS mortality due to drug overdose or other acute and chronic diseases [33].

Model outputs include age- and sex-specific data on the number of PLHIV, people newly infected with HIV infections, and AIDS related deaths, among other variables.

Country files are developed by country HIV estimates teams and are sent to UNAIDS for review and compilation. This process is reversed for a number of high-income countries where files are produced by UNAIDS and countries review the results.

### Projection of PHIV50+ estimates

To project PLHIV50+ for 2017–2020, the Scenario Generator tool within Spectrum was used to project the course of the epidemic given specific programmatic targets such as adults on ART. Following the UNAIDS 90–90–90 treatment target [34], and in line with country aspirations under the 2016 Political Declaration on HIV, projections for 2017–2020 were calculated with Spectrum version 5.57, which is a slightly updated version than that used for the historical estimates and based on an assumed scale-up of ART to reach 81% coverage by 2020 for each country with available Spectrum files, i.e. for 160 countries. Incidence was assumed to follow the current trajectory adjusted for the reduction in transmission among people receiving ART. Spectrum assumes a 70% reduction in infectivity among persons on ART. Uncertainty bounds around PLHIV50+ are based on uncertainty bounds for PLHIV aged 15 years and over, calculated in Spectrum.

## Results

**Fig 1:**
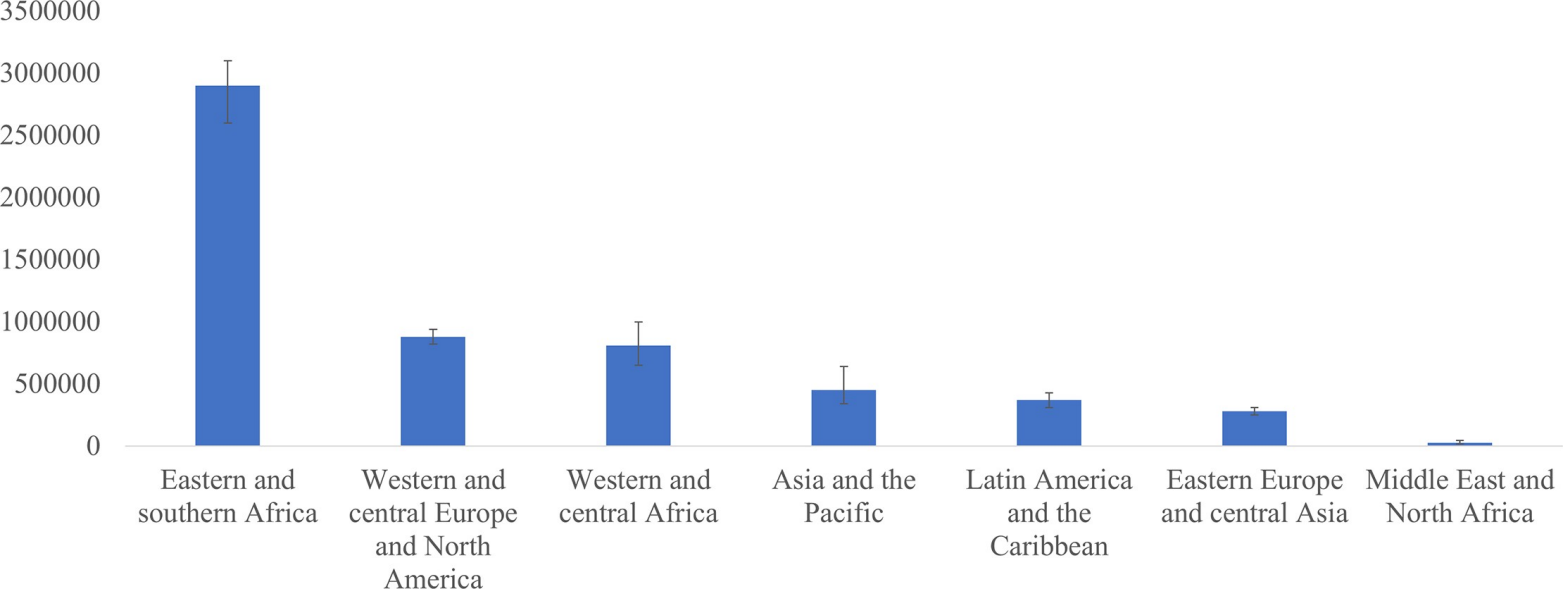
Number of people living with HIV who are aged 50 years and older, by region, 2016.

In 2016, there were an estimated 5.7 million [4.7 million–6.6 million] PLHIV50+ globally. The number of PLHIV50+ has nearly tripled since 2000 (S1 Table). This number is expected to increase to an estimated 7.5 million [6.3 million–8.8 million] by 2020 provided that countries meet the UNAIDS treatment target of 81% ART coverage. Eastern and Southern Africa is the region primarily affected with an estimated 2.9 million PLHIV50+ [2.6 million–3.1 million] in 2016, more than three times the PLHIV50+ in Western and Central Africa, or Western and Central Europe and North America (Fig 1).

**Fig 2:**
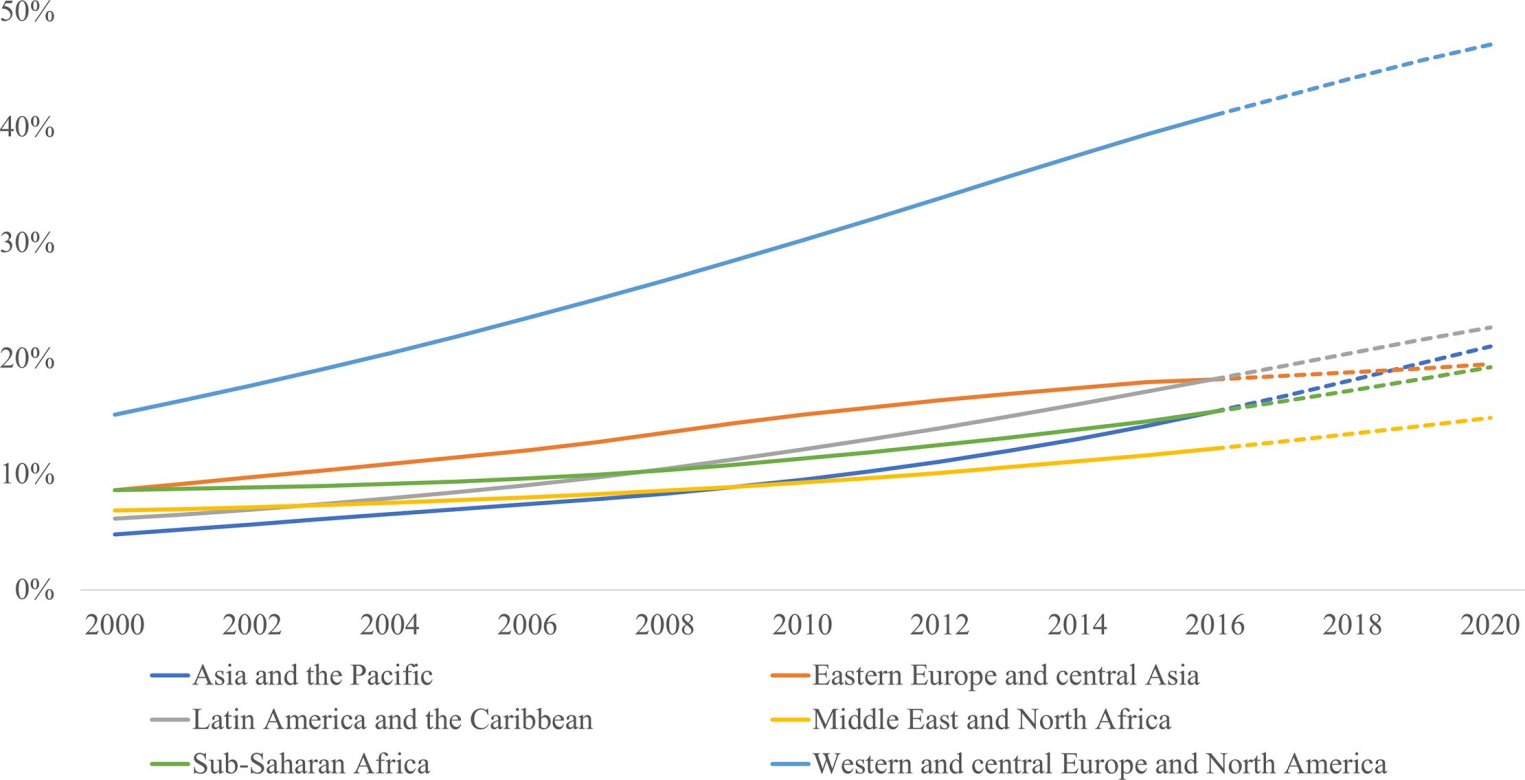
Proportion of people living with HIV who are aged 50 years and older, by region, 2000–2020.

The ageing of the global HIV epidemic is also reflected in the proportion of PLHIV50+ that has increased from 8% in 2000 to 16% in 2016, a proportion that is expected to reach an estimated 21% by 2020. These proportions in 2016 have more than doubled in Eastern and Southern Africa, Eastern Europe and Central Asia, and Western and Central Europe and North America since 2000; they have tripled in Latin America and the Caribbean, and quadrupled in Asia and the Pacific regions (Fig 2.).

When disaggregated by 10-year age groups, the 50–59 year old PLHIV group comprises the greatest proportion of PLHIV50+, ranging from 62% in Western and Central Europe and North America to 77% in the Middle East and North Africa Region, reflecting the growing populations in the latter region. Regional differences in the male-to-female ratios by age group were observed in 2016: considerably more men aged 50+ were living with HIV compared to women in all regions except for the sub-Saharan African regions, where the male-to-female-ratio was 0.9 (Table 1).

**Table 1:** Expected proportion of people living with HIV aged 50 years or older among all people living with HIV and male-to-female ratio in the year 2016, by region.

		Eastern & Southern Africa	Western & Central Africa	Western &Central Europe & North America	Asia & the Pacific	Latin America & the Caribbean	Eastern Europe & Central Africa	Middle East & North Africa
PLHIV 50+ / PLHIV Total		14.7%	13.2%	40.9%	8.9%	17.8%	18.1%	11.7%
Male to female ratio	> = 50 years	0.83	1.10	4.12	3.00	2.39	1.73	2.72
	<50 years	0.66	0.73	2.87	1.65	1.81	1.26	1.54

**Fig 3:**
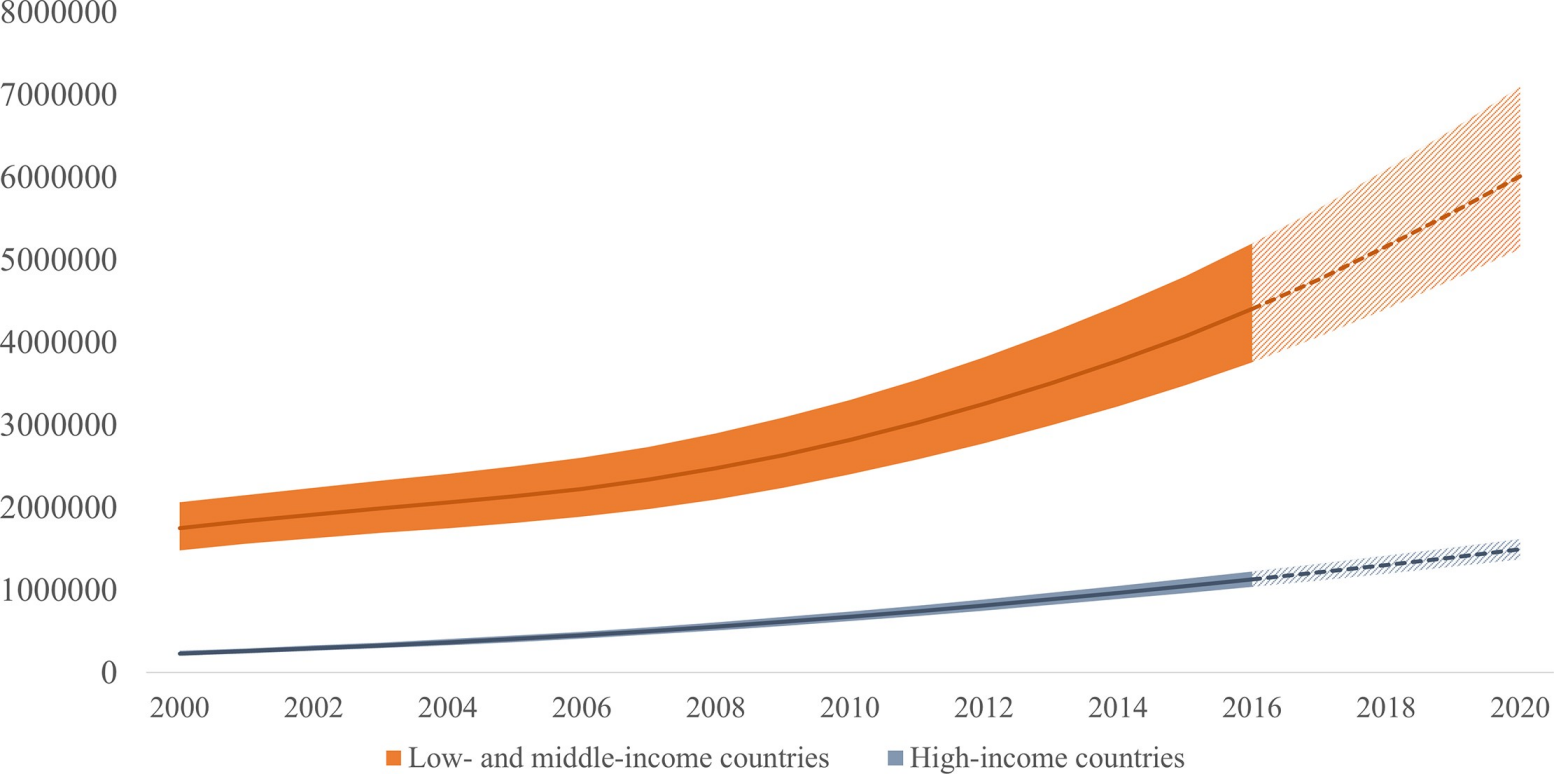
Number of people living with HIV who are aged 50 years and older, high-income countries, and low- and middle-income countries, 2000–2020.

**Fig 4:**
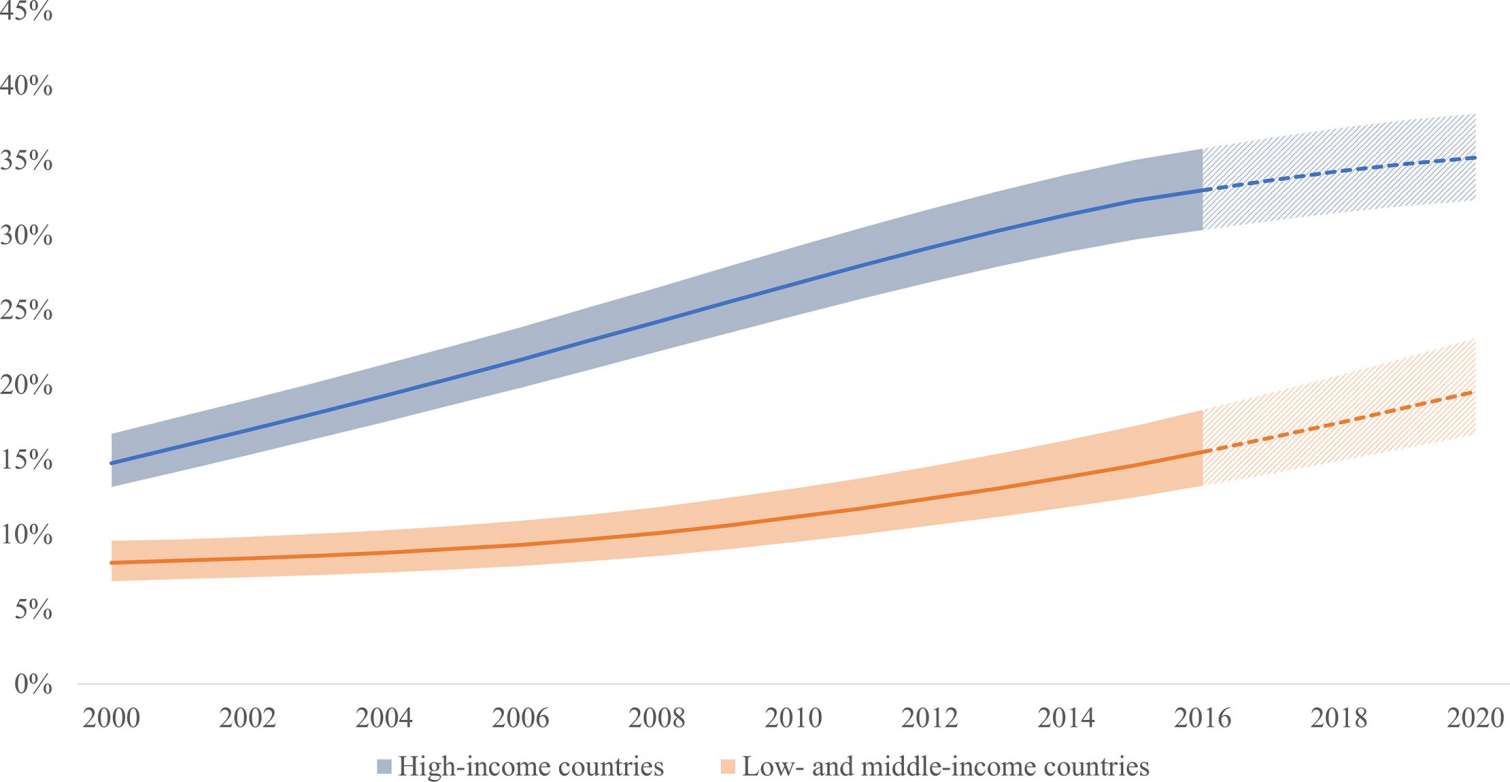
Proportion of people living with HIV who are aged 50 years and older, high-income countries, and low- and middle-income countries, 2000–2020.

In 2016, 80% of PLHIV50+ lived in low-and middle-income countries (LMICs). The number of PLHIV50+ in LMICs increased from 1.8 million in 2000 to 4.5 million in 2016, and is estimated to increase to 6.5 million by 2020. Between 2000 and 2016, the proportion of PLHIV50+ in LMICs increased from 7% to 14%, compared with an increase from 15% to 33% in high income countries (HICs) for the same time period. (Figs 3 and 4).

In 2016, an estimated 110,000 [93,000–120,000] people aged 50+ were newly infected across the world, of which 79% were living in LMICs and 64% resided in sub-Saharan Africa. Out of the global number of AIDS-related deaths among PLHIV50+, 60,000 [49,000–71,000] in 2016, 90% occurred in LMICs, with 69% in sub-Saharan Africa, and 11% in the Asia-Pacific region (S1 Table).

## Discussion

The proportion of PLHIV50+ around the world increased substantially from 8% in 2000 to 16% in 2016 and is expected to reach an estimated 21% by 2020. In 2016, 80% of PLHIV50+ lived in LMICs and this percentage is expected to continue to increase. Eastern and Southern Africa is home to the largest number of PLHIV50+, however, all regions around the world have substantial numbers of PLHIV50+ and all are likely to increase by 2020.

The increases are seen among PLHIV50+ that were infected before the age of 50 and those infected after the age of 50. The greater proportion of PLHIV50+ living in LMICs are due to the larger number of PLHIV living in these countries and the global roll-out of ART that has been recently observed [27]. Currently, a higher proportion of the PLHIV living in HICs are aged older than 50 years, but these proportions are also increasing in LMICs. The current difference between HICs and LMICs is not only a reflection of the larger number of PLHIV living in LMICs but also due to the delayed roll-out of ART in LMICs compared with HICs. As the roll-out of ART in LMICs continues at a rapid pace, partly attributable due to the 90-90-90 Strategy [34], the number and proportion of PLHIV50+ is likely to continue to increase in LMICs.

As PLHIV age, the likelihood of them developing Noncommunicable diseases (NCDs) increases and their overall burden of NCDs will increase in the future. A recent study projected that by 2030, 84% of Dutch PLHIV will be suffering from at least one NCD in addition to HIV [16]. The underlying pathophysiological cause is chronic inflammation by chronic immune activation. PLHIV have higher markers of inflammation [35] and HIV treatment failure increases with increased inflammation [36]. The process of chronic inflammation is further accentuated by other chronic diseases, as well as socio-economic and behavioural factors [37].

Currently, the corner-stone of the clinical management of PLHIV50+ remains treatment with ART complemented by the treatment of opportunistic illnesses, other infections and the treatment of non-HIV comorbidities. Some of the chronic conditions seen at increased prevalence among PLHIV include myocardial infarction [38], stroke, diabetes, renal disorders [39], osteoporosis [40], frailty [41], neurocognitive decline and non-AIDS defining malignancies [40,42]. PLHIV are twice as likely to develop cardiovascular disease and because of the prevalence of HIV in LMICs, the population attributable fraction for CVD is highest in LMICs, especially in Eastern and Southern Africa [43]. Apart from its therapeutic effect, ART also reduces HIV transmission [44].

Few educational programs, campaigns and messages in LMICs have profiled the risk of HIV transmission among older people and they are therefore often less aware than younger people about the risk of acquiring or transmitting HIV and may engage in risky sexual activities [45,46]. Most HIV-related information and prevention messaging are tailored at younger people resulting in lower levels of HIV-related awareness and knowledge among older adults [47]. The few population-based surveys that have been conducted among people aged 50 and over, were primarily focused on men [48]. Older people need tailored messages and programming that emphasize engagement in communities and programmes, empowerment, and achievement of better health outcomes through combining biomedical, behavioral, and structural approaches [49]. Cultural factors may inhibit younger healthcare providers in LMICs from discussing such issues with older patients [50] and such barriers may be contributing to lower HIV testing uptake, especially among older women [51].

PLHIV50+ have a greater burden of co-morbidities and treatment side effects compared with younger PLHIV. In some communities, PLHIV50+ may have better adherence levels [24], but this is often dependent on the social support in their respective communities. Misconceptions are also common regarding sexual activity among older people, which limits the development of appropriate responses for people aged 50 and older, irrespective of HIV-serostatus.

PLHIV and other key population groups are marginalized, stigmatized and discriminated against, and face a range of health and social challenges [52,53]. PLHIV50+ are more likely to be single, live alone, have fewer friends and lack adequate social support networks compared to younger PLHIV [54,55]. This may result in less available community support for PLHIV50+, while they possibly also face additional stigma from being ‘old’, resulting in additional isolation and decreased social support [56]. It is important for healthcare providers to be aware of the increasing number of PLHIV50+, many of whom may require the use of geriatric care [57].

To deliver ‘seamless’ healthcare in a country, HIV services and non-HIV services need to be linked at institutional levels and integrated at the level of service provision. As the number of PLHIV50+ increases, requirements for integrated services will also increase [58]. To monitor the linkage of HIV and non-HIV services, countries are developing and implementing national health identifiers [59]. Their implementation needs to be accompanied by effective measures to protect the confidentiality and security of personal health information [60].

The models used in this paper rely on regional and epidemic-specific assumptions about the age distributions of people newly infected with HIV. The uncertainty bounds around the estimates capture some of this imprecision. Similarly, the age distribution of people receiving ART are not available from most countries and thus the models assume that the age distribution of people initiating ART is similar to the age distribution of people eligible for ART. The underlying non-AIDS mortality to people living with HIV in the estimates is assumed to be similar to the general population. Future additional research and data are expected to improve those assumptions in the coming years.

### Conclusion

The number of PLHIV50+ is increasing worldwide, with the largest numbers observed in LMICs. Any person diagnosed with HIV should be started on ART as soon as possible. As people in this age group are sexually active, they will also need to be reached through HIV prevention campaigns, and HIV testing and counselling programs. Care givers need to be aware of the changing physical, psychological and social needs of ageing in PLHIV50+ and need to ensure optimum psychological and social support where required [61,62]. Increased surveillance and patient monitoring need to be focused on PLHIV50+ and agencies reporting HIV-trends should routinely analyze trends occurring in PLHIV50+. At country level, HIV and non-HIV services need to be linked more strongly as PLHIV50+ are the group par excellence that will increasingly need the use of non-HIV services.

## Supporting information

S1 Table Global and regional trends of estimates for people living with HIV aged 50 years and older, 2000–2016. (XLSX)
